# Silencing of Aquaporin Homologue Accumulates Uric Acid and Decreases the Lifespan of the Asian Citrus Psyllid, *Diaphorina citri* (Hemiptera: Liviidae)

**DOI:** 10.3390/insects12100931

**Published:** 2021-10-13

**Authors:** Yulica Santos-Ortega, Nabil Killiny

**Affiliations:** 1 Department of Plant Pathology, Citrus Research and Education Center, IFAS, University of Florida, 700 Experiment Station Road, Lake Alfred, FL 33850, USA; yulica.santosortega@usm.edu; 2 Department of Biological Environmental and Earth Sciences, University of Southern Mississippi, 118 College Drive, Hattiesburg, MS 39406, USA

**Keywords:** Asian citrus psyllid, *Diaphorina citri*, RNA interference, gene silencing, aquaporin, uric acid

## Abstract

**Simple Summary:**

The use of RNA interference has become increasingly popular for investigating insect physiology, testing the functionality of insect genes and as a potential control strategy. Hemiptera include many vectors for destructive plant diseases. A major characteristic of the order of Hemiptera is feeding on the phloem sap of their plant hosts. Phloem feeders face high osmotic stress between the gut lumen and hemolymph due to the high level of sucrose in phloem sap. Targeting the osmoregulation mechanisms in Diaphorina citri Kuwayama, which transmits ‘Candidatus Liberibacter asiaticus’, the putative causal agent of Huanglongbing in citrus may lead to an effective control strategy. Herein we downregulate the expression of aquaporin, representing a major mechanism of osmoregulation, by RNA interference.

**Abstract:**

The Asian citrus psyllid*, Diaphorina citri* Kuwayama is devastating the citrus industry worldwide. It transmits ‘*Candidatus* Liberibacter asiaticus’, the pathogen of Huanglongbing in citrus. RNA interference is an excellent tool for functional genomics and for screening target genes for pest control. Herein, we silenced the aquaporin (AQP) gene (*DcAQP*) homologue in *D. citri* to study its functionality and whether it could be a good target for a control strategy. AQP is an integral membrane channel protein that aids in the rapid flux of water and other small solutes that move across the lipid membrane. In Hemiptera, it is well established that AQP plays important roles in adjusting to physiological challenges including (1) regulating osmotic stress between the gut lumen and hemolymph after imbibing large quantities of a low nitrogen, sugar-rich liquid diet; (2) avoiding or preventing dehydration and desiccation; and (3) surviving at elevated temperatures. The dsRNA-*DcAQP* was applied twice to nymphs of the 4th and 5th instars through a soaking technique. Silencing AQP caused a significant increase in nymph mortality. Emerged adults showed malformations and a shorter lifespan. Silencing *DcAQP* provoked alterations in some metabolites and increased the uric acid content in emerged adults. *DcAQP* could be a useful target to control *D. citri*.

## 1. Introduction

Aquaporins (AQPs), as the name denotes, are specialized integral membrane proteins that form pores through which water and small neutral molecules are selectively channeled across cellular membrane [1]. Ubiquitous in nature, AQPs belong to a large class of proteins called major intrinsic proteins (MIPs) and play critical roles in osmoregulation [2,3]. AQPs have garnered great interest and have been intensively studied since the first description of aquaporin from red blood cells [4]. Two regions are distinctive of the family of AQPs: the NPA-region (Asn-Pro-Ala), found in the middle of the channel, and the aromatic–arginine (ar/R) region located on the extracellular side of the cell membrane. The NPA-region is substrate permeable and consists of two Asn-Pro-Ala motifs embedded in the hydrophobic interior of the membrane channel [5,6]. The Ar/R-region serves as a selectivity filter for water and neutral solutes, facilitating water transport by restricting larger molecules from entering the channel from the extracellular sides of the membrane [1,2,7,8,9]. AQPs have been classified into three subfamilies: the orthodox or traditional water-selective aquaporins (AQP0, 1, 2, 4, 5, 6 and 8); glycerol-transporting aquaglyceroporins (GLPs), which may also transport small non-charged solutes like urea and metalloids (AQP3, 7, 9 and 10); and unorthodox or super aquaporins (AQP11 and 12), which contain 1 NPA motif and one variant motif and are found only in animal cells [10].

The AQPs form water channels through the plasma membrane of the cell in order to passively transport water in response to osmotic gradients, and they have an important role in cell volume regulation and excretion [1]. Specifically in insects, the AQPs are indispensable for cellular water regulation, and their presence is an adaptation to survive in extreme environmental conditions such as subzero temperatures and drought. In these cases, water removal from cells is critical to initiating diapause or hibernation and for avoiding damage from ice crystal formation [11,12]. Other insects need the AQPs to cope with diets consisting of excess liquids such as blood or plant saps (phloem and xylem) [11].

Insects belonging to the order Hemiptera ingest large quantities of plant sap to acquire sufficient levels of nitrogen and other nutrients. Plant sap contains primarily water and sugars as main components, which provoke the increase of osmotic pressure in the insect gut. In order to overcome this challenge, the insects quickly break down sucrose into fructose and glucose, which are transported to the interior of the cells, and the remaining sugars in the gut are oligomerized and eliminated as honeydew [13,14,15]. Aquaporin water channels in the gut of many insects may serve as the first opportunity to reduce the osmotic pressure induced by phloem feeding by allowing water to pass through the plasma membranes from the hemolymph into the gut lumen [15,16]. The survival of *D. citri* and other hemipterans depends on their ability to osmoregulate after feeding on plant phloem sap, which is known for its rich sucrose content, but such feeding may increase the osmotic pressure up to 4–5 times [13]. In fact, Douglas [15] noted “phloem-feeding insects are expected to shrivel as they feed” if not for the ability to reduce their osmotic pressure. The primary mechanism for maintaining osmotic pressure in *D. citri* is by sucrose hydrolase activity in the gut [17]. Other works have identified and characterized AQP activity as a secondary mechanism that facilitates the osmotic movement of water from the distal gut to the proximal midgut (stomach) and promotes pressure equilibrium between the gut lumen and the hemolymph. The works with *Bemisia tabaci* and *Acyrthosiphon pisum* mentioned that the silencing of AQP1 provoked disruption of osmoregulation due to a significant elevation in the osmotic pressure [16,18].

Some Hemipterans such as leafhoppers, froghoppers, whiteflies, and psyllids have a characteristic water-conducting anatomical complex called the filter chamber that bypasses part of the digestive tract and functions to shunt rapidly and directly large amounts of fluids from the foregut to the midgut [19]. This structure, together with the AQPs, participates in water cycling in the alimentary tracks of sap-feeders and helps to alleviate osmotic pressure. In fact, the AQPs are localized in the filter chamber of *Cicadela viridis* and in the anterior ileum of the hindgut of *B. tabaci* [18,20] where they act to remove excess dietary water [21]. In other phloem-feeding insects such as aphids that lack a filter chamber, the close proximity between the distal and proximal regions of the gut facilitates water transfer [16]. AQP1 was immunolocalized in the distal intestine, and its probable function most likely relates to shunting water to the stomach to relieve osmotic stress [16].

Owing to their high specificity, RNA interference (RNAi) techniques to silence genes have been widely accepted as an alternative method to control insect pests of importance to human health and agriculture [22,23]. Therefore, AQPs present a worthy target for insect pest management through RNAi technology. The silencing of *ClAQP* in the human bed bug *Cimex lectularius*, which transmits *Trypanosoma cruzi*, severely affects its excretion, water homeostasis and reproduction [22]. The fitness of the tick, *Rhipicephalus* (*Boophilus*) *microplus,* which transmits the protozoan *Babesia bovis,* was decreased by silencing of *RmAQP2* [24]. In the pea aphid, the silencing of *ApAQP1* caused significant elevation in osmotic pressure of the hemolymph [16]. Recently, in the potato psyllid *Bactericera cockerelli*, Ibanez, Hancock and Tamborindeguy [10] found that AQPs play a role in sustaining the primary endosymbiont and, consequently, could be a valuable target for studying this interaction.

*Diaphorina citri* Kuwayama (Hemiptera: Liviidae) is one of the most notorious agricultural pests because it transmits and spreads the bacterium ‘*Candidatus* Liberibacter asiaticus’ that causes huanglongbing (HLB) or citrus greening disease [25,26]. HLB disease is devastating for the citrus industry, and the immediate solution to controlling transmission has been through managing the vector, *D. citri*. However, despite efforts to reduce the population of insects with insecticides and biological controls, these measures have not been sufficient [27,28]. In *D. citri*, RNAi techniques have proven to be a powerful tool to assess the efficacy of genes targeted to reduce psyllid survival and fecundity, increase susceptibility to insecticides, increase mortality, cause abnormal conditions, or interfere with homeostasis [29,30,31,32,33].

AQP is one of three mechanisms insects use to regulate osmotic pressure. In addition to AQP, sucrose hydrolase acts to break down sucrose into fructose and glucose, while sucrose transglucosidase oligomerizes glucose into long-chain oligosaccharides, primarily trehalose, which is excreted as honeydew [15]. These three mechanisms act together to reduce high osmotic pressure caused by the ingestion of a liquid diet high in sucrose. In our previous work, we silenced sucrose hydrolase (*DcSuh*) in *D. citri* using RNAi, which resulted in disruption of the osmotic homeostasis in *D. citri* [17]. This led us to investigate other candidate genes related to water balance, such as aquaporins. We used the databases to identify the aquaporin gene of *D. citri* using in silico analysis and silence the *AQP* gene in *D. citri* by means of RNAi technique. We hypothesize that silencing *DcAQP* using dsRNA will result in knockdown of its transcript expression, which will cause increased mortality in adults, owing to their inability to maintain internal water homeostasis.

## 2. Materials and Methods

### 2.1. Diaphorina citri Colonies

Laboratory colonies of *D. citri* were continuously reared on 6-month-old *Citrus macrophylla* (Alemow) plants with new flush and enclosed in insect rearing cages (60 × 60 × 90 cm^3^, Bioquip). The *D. citri* colonies were maintained in temperature-controlled growth rooms set at 25 ± 3 °C, 60 ± 5% relative humidity (RH) and with a 16:8 h (light:dark) photoperiod. The citrus and insect cultures were located at the Citrus Research and Education Center (CREC), University of Florida, Lake Alfred, FL, USA.

### 2.2. In Silico Analysis

The aquaporin sequences were queried from *D. citri* Genome and Transcriptome https://citrusgreening.org/organism/Diaphorina_citri/genome (accessed on 10 September 2021). Several AQPs sequences from the databases were compared with the information in NCBI and UniProt through BLAST with aquaporins well described in other insects. These protein sequences included AQP of *A. pisum* (accession no. NP_001139376.1), *B. tabaci* (accession no. XP_018906592.1; ABW96354.1) and *Myzus persicae* (accession no. A0A0H3XRM3) from Hemiptera and *Aedes aegypti* (accession no. Q9NHW7.2) and *D. melanogaster* (accession no. NP_001286316.1) from Diptera. Multiple amino acids sequence alignment was performed using Clustal W and Boxshade 3.21. to visualize conserved regions.

Membrane topology and domain structure predictions of the *DcAQP* protein sequence were examined with TMHMM and SMART. The theoretical isoelectric points (pI) and molecular weights (MW) of protein were computed using the Compute pI/MWTool (http://web.expasy.org/compute_pi/, accessed on 10 September 2021). The SWISS-MODEL and GENO 3D servers were used to predict the secondary structure to validate template selection, and it was visualized using the PyMOL (www.pymol.org, accessed on 14 September 2021). The phylogenetic relationship was performed by MEGA7 using the neighbor-joining method with 1000 bootstrap replicates to evaluate the significance of the nodes.

### 2.3. Double-Stranded DNA Synthesis

The *D. citri* predicted aquaporin AQPA (*DcAQP*), mRNA is 815 bp in length (NCBI-GenBank: XM_008486010.2). Primers were designed from the open reading frame sequence (ORF) using the Primer Plus 3 program, which considers several parameters including CG content (50–55%), melting temperature (59.95–60.01 °C), product length (300–401 bp) and undesirable alignments. A primer pair *DcAQP*-F and *DcAQP*-R was generated to obtain a 306 bp DNA fragment through gBlocks^®^ Gene Fragments (Integrated DNA Technologies, Coralville, IA, USA), and all primer sequences used in this work are presented in Table 1. Then, the DNA fragment was amplified with the T7 promoter by PCR (primers: *DcAQP*T7 F-R) and used as the template to generate dsRNA with the MEGAscript RNAi kit (Ambion). We synthesized the dsRNA-*GFP* (primers: gfpT7 F-R) following the same procedure mentioned above for use as a negative control. The quantity and integrity of synthesized dsRNA was evaluated by a NanoDrop 1000 spectrophotometer (Thermo Fisher Scientific, Waltham, MA, USA) at 260 nm and by agarose gel electrophoresis.

### 2.4. Administering of dsRNA

The nymphs of *D. citri* were removed from the leaves and branches of *C. macrophylla* plants with a #2 camel hairbrush. Then, nymphs were examined under a stereomicroscope and classified into 1st through 5th instars based on morphological features described in [35]. Combining the 4th and 5th nymphal instars for some assays provided ample biological material without affecting the overall outcomes of the study. Serial concentrations of dsRNA-*DcAQP* were prepared in RNase-free water as follows: (ng·µL^−1^) 25, 50, 75, 100, 200, 500 and 1000. In addition, RNase-free water and dsRNA-*GFP* were used as negative controls. Third through fifth instar nymphs were starved for 2 h and then soaked with a 10 μL dsRNA droplet, and the application was repeated after 24 h. The dsRNA droplet was withdrawn by a pipette after soaking for about 10 min. After dsRNA applications, treated nymphs were placed on filter paper to dry, and then were transferred onto small *C. macrophylla* plants housed in modified Berlese funnel traps (funnel removed).

### 2.5. Gene Expression Using Semi-Quantitative RT-PCR

For *DcAQP* transcript fragment evaluation by semi-quantitative RT-PCR, 2 h after the second dsRNA application, the nymphs were washed with RNase-free water to removed dsRNA-*DcAQP* residues and were processed. A primer pair (*DcAQP*SRT F-R) was designed to be inside the dsRNA-*DcAQP* trigger sequence for semi-quantitative RT-PCR analysis, which produced an amplimer of 234 nucleotides. Then, total RNAs were extracted in TRIzol^®^ Reagent (Invitrogen) from untreated eggs, nymphs in first, second, third, combined fourth-fifth stages, and adults using a Direct-zol^TM^ RNA MiniPrep kit (Zymo Research, Irvine, CA, USA). Total RNA (100 ng) from each sample was used to amplify the transcript fragments of *DcAQP*, and actin was used as an internal control (GenBank Accession number: DQ675553, Reference Primer ActRT F-R) with the Affinity Script One-Step RT-PCR kit (Agilent). Reactions were subjected to the thermal program: 45 °C for 5 min, 92 °C for 1 min; 30 cycles of 92 °C for 20 s, 57 °C for 20 s and 72 °C for 30 s; followed by maintenance at 72 °C for 3 min. PCR products were analyzed on a 1.5% agarose gel (Thermo Fisher Scientific) and visualized by GelRed staining (Biotium, Fremont, CA, USA).

### 2.6. Gene Expression Using Quantitative PCR

To evaluate the effect of dsRNA-*DcAQP* on aquaporin gene expression, we treated nymphs with 200 and 500 ng/µL dsRNA such as mentioned above using the same soaking protocol, and the application was repeated after 24 h. After adults emerged, total RNA was isolated from the whole bodies of adult *D. citri*. The primers used for qRT-PCR analysis (*DcAQP*RT F-R) were used at 125 nM. First-strand cDNA synthesis was initiated with 0.6 µg of purified RNA using SuperScript IV VILO Master Mix (Thermo Fisher, Waltham, MA, USA). The synthesized cDNA was diluted 1:5 (10 ng/µL working concentration) and stored at −20 °C until use. The quantitative RT-PCR reactions were carried out in a 20 µL mixture consisting of 10 µL of Power Up SYBR Green master mix (2×) (Thermo Fisher, Waltham, MA, USA), 1 µL of primer pair mix, 2 µL of cDNA and 7 µL of RNase-free water. All the samples were placed in QuantStudio^TM^ 3 Real Time PCR detection system (Applied Biosystems, Waltham, MA, USA). One reference gene, actin (Genbank accession number DQ675553) was used to normalize the amount of cDNA added to the PCR reactions.

### 2.7. Insect Bioassays

#### 2.7.1. Mortality and Survival Assay

For mortality studies, three replicates per treatment consisting of 20 *D. citri* nymphs each (from 4th and 5th instars) were treated with dsRNA-*DcAQP* at the concentrations mentioned above. The mortality rate was calculated based on the numbers of emerged adults 7 d after treatment. Experimental data from mortality assays were carried out with JMP software, using one-way ANOVA, and multiple comparisons between treatments were performed with the Tukey’s Honestly Significant Difference (HSD) test. For the survival assay, we recorded the numbers of adults dead daily, and the analysis was performed with JMP software using the Kaplan–Meier method. Statistical significance was established as *p* < 0.05. During the experiment, the dead or dying *D. citri* were examined morphologically under a stereomicroscope. The psyllids with malformations were photographed using a Canon Power Shot S3IS digital camera coupled to a M3Z stereomicroscope (Leica).

#### 2.7.2. Body Length and Weight

Three biological replicates (20 nymphs per treatment) were treated with 0, 25, 100, 200, 500 and 1000 (ng·µL^−1^) dsRNA-*DcAQP*. The adult body length measurements were taken after 10 days with the emerged adults. We made two measurements: (A) from the head to the end of the wings, and (B) from the head to where the body ends. We calculated the average body weight of the emerged adults by dividing the total weight of living adults by the number of adults. We statistically analyzed the experimental data from weight and body length measurements with SigmaPlot software, one-way ANOVA, and Tukey’s HSD test for multiple comparisons between treatments.

### 2.8. Metabolomic Assay

#### 2.8.1. Collection and Extraction of *D. citri* Metabolites

To determine the effects of silencing *DcAQP* on *D. citri* metabolites, we carried out two separate GC-MS protocols on adult *D. citri* emerged from treated nymphs. We previously standardized the following two methodologies in our laboratory: N-methyl-(N-trimethylsilyl) trifluoracetamide (MSTFA) [36] and methylchloroformate (MCF) [37]. We use the two methods together because they complement each other in the types of compounds that they detect. Briefly, for MSTFA and MCF analyses, we collected groups of 50 and 100 adult *D. citri*, respectively, after two applications (24 h apart) of dsRNA-*DcAQP* at 200 ng/µL as nymphs and immediately placed them into 1.5 mL microcentrifuge tubes with the appropriate extraction solvent for each derivatization (MSTFA: 150 µL of 8:1:1; methanol: chloroform: water; MCF: 300 µL of 80:19.9:0.1; methanol: water: 6N HCl), and kept them at −20 °C until analysis. A glass bead was added to each tube, and the tubes were cooled in liquid nitrogen for 10 min. Thereafter, the insects were macerated by homogenizing in a TissueLyser II (Qiagen, Hilden, Germany) in the extraction solution for 2 min. Samples were then placed on an Enviro-Genie (Model SI-1200, Scientific Industries, Bohemia, NY, USA) at rotate/rock 20/40 overnight at 5 °C. After the overnight extraction, the tubes of insect homogenate were vortexed briefly and centrifuged at 5 °C for 20 min at 12,000 rpm in a refrigerated centrifuge (Model 5430 R, Eppendorf, Hamburg, Germany) to remove solid debris. The supernatant was collected from each tube and placed into new 1.5 mL tubes. Two biological replicates were made per methodology.

#### 2.8.2. MSTFA Derivatizations

For the first GC-MS analysis using MSTFA derivatization, we placed 100 µL aliquots of adult *D. citri* supernatant into silanized GC vials with 200 µL fused-inserts (MSCert4000–30LVW, National Scientific, Claremont, CA, USA) along with 10 µL of internal standard (1000 ppm ribitol in water, Sigma Aldrich, St. Louis, MO, USA). As a blank reference sample, we derivatized 100 μL extraction solvent plus 10 μL ribitol IS added. The extracted samples and blank samples were dried under a nitrogen stream before adding the derivatization reagents. Methoxyamine hydrochloride solution (MOX) in pyridine (2%) and N-methyl-(N-trimethylsilyl) trifluoracetamide (MSTFA) were purchased from ThermoFisher Scientific. To each of the dried samples, 30 µL MOX was added and incubated at 60 °C for 1 h. Finally, 80 µL MSTFA was added and incubated an additional 1 h at 60 °C. The derivatized samples were injected splitlessly into the GC injector (0.5 µL) for analysis using the same GC-MS column and conditions previously described by Killiny [38].

#### 2.8.3. MCF Derivatizations

For the second GC-MS analysis using methylchloroformate, we transferred aliquots (100 µL) of adult *D. citri* supernatant to 1-mL silanized GC-MS inserts and concentrated them to approximately 20 µL under a nitrogen stream. To the concentrate, we added 180 µL of NaOH (1 M). The alkaline sample was mixed with 167 µL of methanol and 34 µL of pyridine, followed by the addition of 20 µL of MCF. The sample was vigorously mixed for 30 s. An additional 20 µL of MCF was added, and the sample was mixed for another 30 s. A 100 µL aliquot of chloroform was added with vigorous mixing for 10 s, followed by 200 µL of sodium bicarbonate (50 mM) with vigorous mixing for 10 s. The upper layer was discarded, and approximately 100 µL of the organic layer was transferred to a new insert. A few milligrams of sodium sulfate were added to dry the organic layer, and 1 µL was injected into the GC-MS. The GC-MS conditions for MCF derivatizations were as reported previously [36].

## 3. Results

### 3.1. BLASTp and Phylogenitic Analysis of Candidate Aquaporin Homologs of D. citri

**Figure 1:**
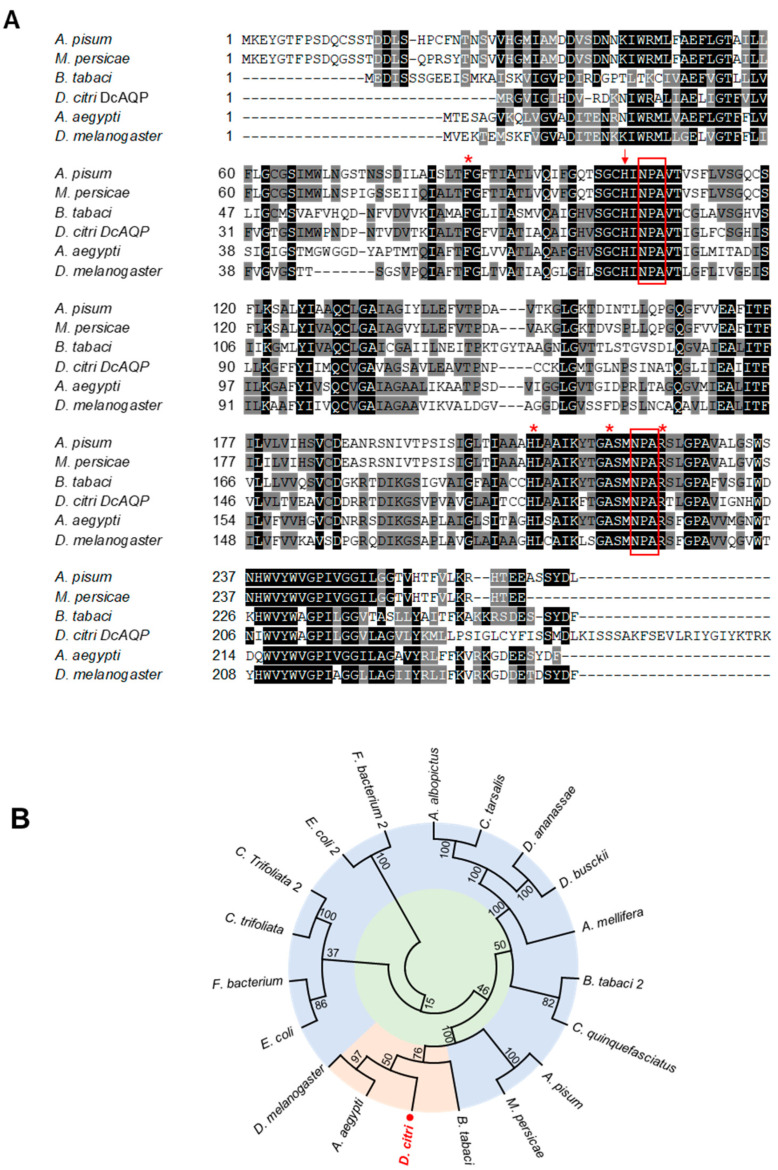
In silico analysis of aquaporin homologue (*DcAQP*) of *Diaphorina citri*. (**A**) Multiple sequence alignment of amino acid sequence of *AQPAe.a* from *D. citri* (*DcAQP*; accession no. XP_008484232.1) and AQPs from other insects, including *A. pisum* (accession no. NP_001139376.1), *B. tabaci* (accession no. XP_018906592.1; ABW96354.1) and *M. persicae* (accession no. A0A0H3XRM3) from Hemiptera and *A. aegypti* (accession no. Q9NHW7.2) and *D. melanogaster* (accession no. NP_001286316.1) from Diptera. Conserved amino acids are indicated with black shading, and those with high similarity score are in gray. The conserved NPA domains are surrounded by red boxes. The AA contributing to the aromatic–arginine (ar/R) region located on the extracellular side of the cell membrane are indicated with red asterisks. (**B**) Evolutionary relationships using an unrooted tree of protein sequences of *DcAQP* from *D. citri* and AQPs from other organisms. The analysis involved 19 amino acid sequences. The *DcAQP* (XP_008484232.1) is colored in red and is marked with a red dot. The full list of genes, names, and accession numbers is available in supplementary Table S2.

In silico analysis using the BLASTp tool showed that the *D. citri* genome possesses several sequences with significant similarity to the aquaporin (*HsAQP1*; accession no. P29972.3) from human, *Homo sapiens* (Table S1). We selected aquaporin AQPAe.a from *D. citri* (*DcAQP*; accession no. XP_008484232.1) as a candidate gene to be targeted by RNAi based on the position of hallmarks of the aquaporin family such as the domains that provide the functionality as a selective water channel, as well as the score of the alignments with *HsAQP1*. The multiple sequence alignment of the AA sequence of *DcAQP* protein with other insects such as *A. pisum*, *B. tabaci* and *M. persicae* (from Hemiptera) and *A. aegypti* and *D. melanogaster* (from Diptera) revealed a high similarity between them (Figure 1A). *DcAQP* shared AA sequence identity of 52.89%, 52.74%, 53.94%, 49.79% and 48.94% with *AQP*s from *B. tabaci*, *D. melanogaster*, *A. aeygpti*, *A. pisum* and *M. persicae*, respectively (Figure 1A). In addition, the phylogenetic analysis revealed that the predicted *DcAQP* was phylogenetically closer to AQPs from Diptera including *A. aegypti* and *D. melanogaster,* followed by species from Hemiptera (*B. tabaci*, *M. persicae* and *A. pisum*) (Figure 1B).

### 3.2. Protein Characterization of Candidate Aquaporin Homologs of D. citri

**Figure 2:**
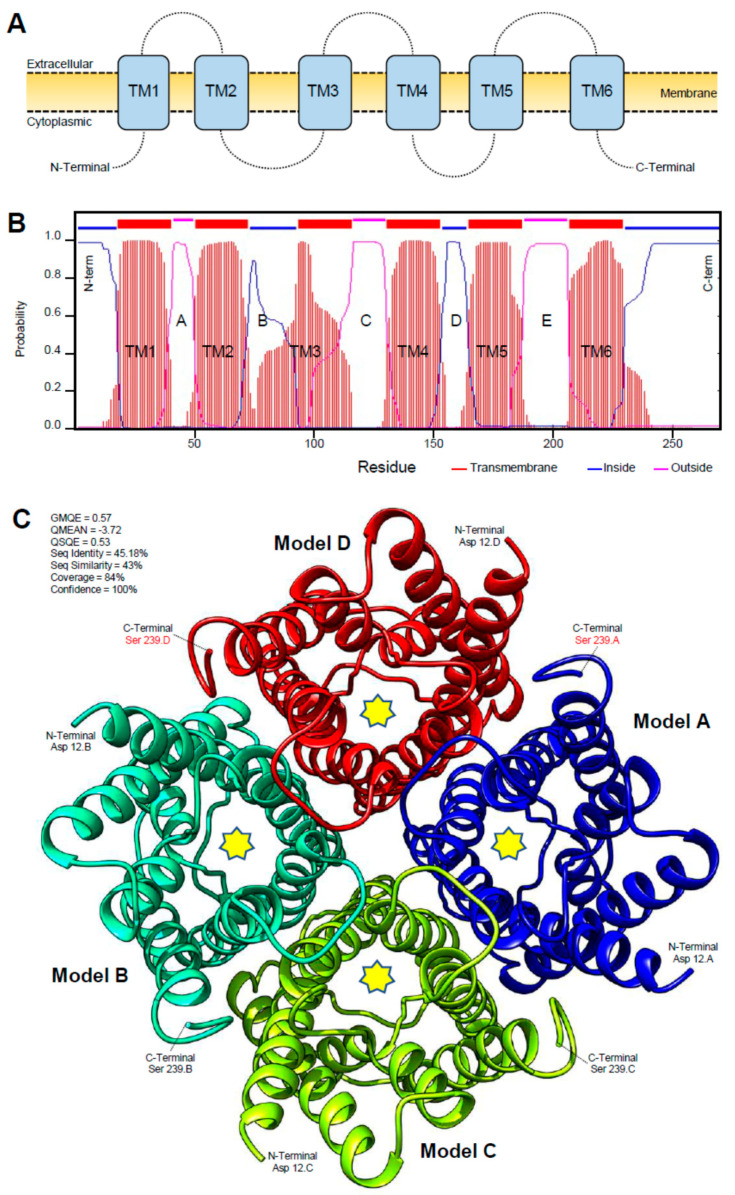
Protein characterization of aquaporin of *D. citri*. (**A**,**B**) Predicted membrane topology of *DcAQP* and domain architecture as predicted by TMHMM Server v. 2.0 and the Simple Modular Architecture Research Tool (SMART), respectively. (**C**) The predicted three-dimensional (3D) secondary model of *DcAQP* using a model from GENO 3D, which used crystallographic coordinates of *Ratus norvegicus* aquaporin 4 in the protein data bank (PDB ID: 2D57). Bioinformatic analyses were carried out using the available data of the official gene set (OGS-v2.0 proteins) for *D. citri* on the citrus greening solutions website (https://citrusgreening.org/organism/Diaphorina_citri/genome) and the available data on the GenBank, National Center for Biotechnology Information website (NCBI, https://www.ncbi.nlm.nih.gov/protein/ accessed on 16 September 2021).

The protein sequence of *DcAQP* consists of 270 AA residues and has a theoretical isoelectric point (pI) of 8.73 and a predicted molecular mass of 28.85 kDa as obtained by pI/MW Tool. The sequence showed two main regions, the canonical signature Asn-Pro-Ala (NPA) localized in B (N, 75; P,76, A,77) and E (N,190; P,191; A,192) loops which are intracellular and extracellular, respectively. Furthermore, the bioinformatic analysis of AA sequences using the TMHMM Server v. 2.0 to predict the transmembrane helices of *DcAQP* and using the Simple Modular Architecture Research Tool (SMART) to predict the domain architecture suggests a high topological similarity between *DcAQP* from psyllid and *HsAQP1* from human. *DcAQP* protein has several key features that are present in a member of the aquaporin superfamily such as the presence of six membrane-spanning helices (TM1 to TM6) separated by five loops with intracellular N- and C-termini (Figure 2A,B).

Although the three-dimensional (3D) structures for arthropod aquaporins are not available yet, we were able to predict the 3D secondary structure of *DcAQP* using a model from GENO 3D, which used crystallographic coordinates of *Rattus norvegicus* aquaporin 4 in the protein data bank (PDB ID: 2D57) (Figure 2C). In Geno 3D, the predicted 3D model energy was of −8275.97 (kcal· mol^−1^), and the similarity between both sequences was 47.5%. The Ramachandra plot indicated that 82.4% of amino acid residues were in the favorable region, 14.3 in the allowed region, 2.7% in the generously allowed region and 0.5% in the disallowed region. Taken together, these findings suggest that the *DcAQP* belongs to the traditional Class 1 of aquaporin, which is mostly water selective.

### 3.3. DcAQP Is Highly Expressed at Late Developmental Stages of D. citri

**Figure 3:**
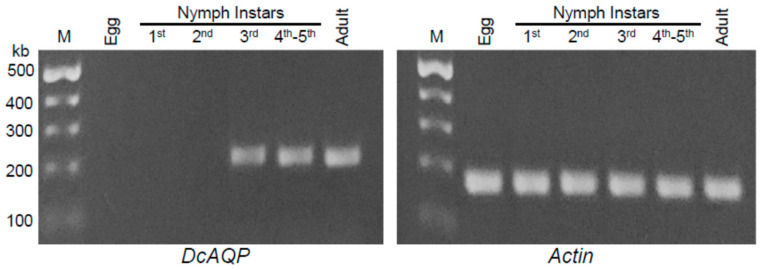
Transcript levels of *DcAQP* in different developmental stages during the life cycle of *D. citri*. Total RNAs were extracted using TRIzol^®^ Reagent from different developmental stages of *D. citri* including eggs and first, second, third, fourth and fifth nymphal instars and adults. After amplification, transcript levels of *DcAQP* were semi-quantified on a 1.5% agarose gel. Actin was used as an internal control gene.

To determine the best stage(s) of the *D. citri* life cycle to target *DcAQP*, we investigated the transcript levels of *DcAQP* throughout the whole life cycle (Figure 3). We found the *DcAQP* transcript highly expressed in the late developmental stages of *D. citri* (third, fourth and fifth nymphal instars) and in adults, but not the early stages (egg and first and second nymphal instars) (Figure 3). All studied stages expressed the constitutive internal control gene, *DcActin.*

### 3.4. Application of dsRNA-DcAQP Reduces DcAQP Expression and Increases the Nymphal Mortality

**Figure 4:**
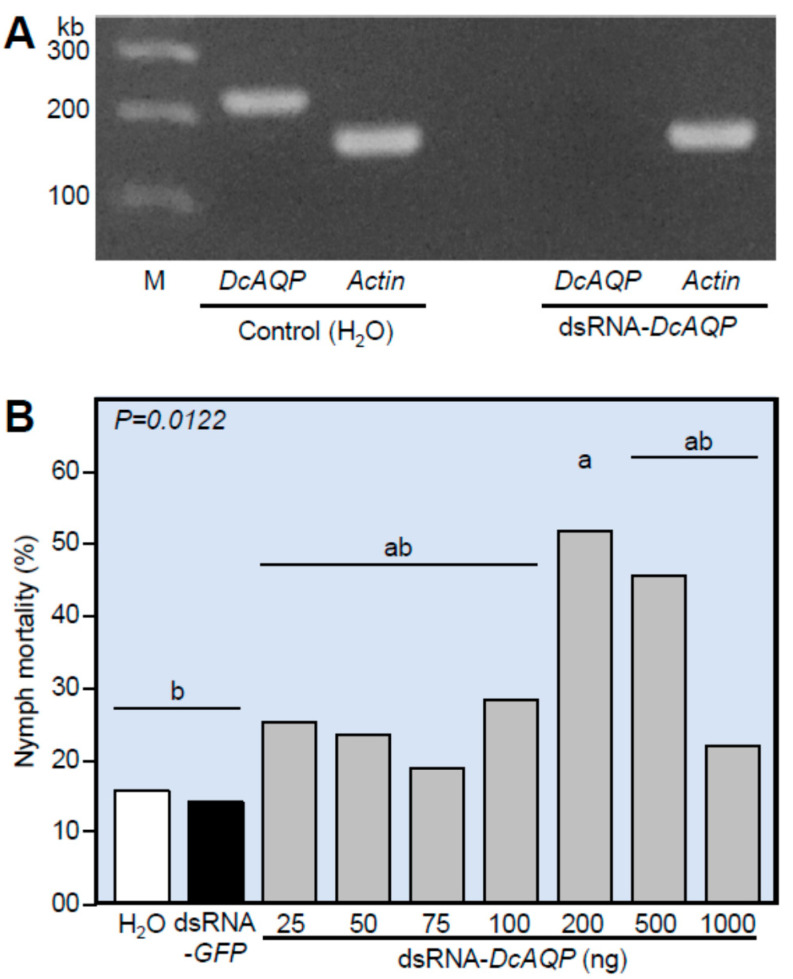
Effect of *DcAQP* silencing on the expression of *DcAQP* and the mortality of *D. citri* nymphs. (**A**) Semi-quantification of *DcAQP* after the treatment with 500 ng of dsRNA-*DcAQP*. Total RNAs were extracted using TRIzol^®^ Reagent from 4th–5th nymph instars treated twice with dsRNA-*DcAQP* in comparison with H_2_O as a control. After amplification, transcript levels of *DcAQP* were semi-quantified on a 1.5% agarose gel. Actin was used as an internal control gene. (**B**) Mortality (%) of 4th–5th nymph instars of *D. citri* treated twice with different concentrations of dsRNA-*DcAQP* (25, 50, 75, 100, 200, 500 and 1000 ng·µL^−1^) in comparison with RNase-free water and dsRNA-*GFP* as controls. Bars and error bars represent means and SDs, respectively. Different letters indicate statistically significant differences among treatments, while the same letter signifies no significant differences among treatments using Tukey HSD (*p* < 0.05).

To test the efficiency of both designed dsRNA-*DcAQP* and the application method, we studied the effect of dsRNA-*DcAQP* on the transcript levels of *DcAQP* in the 4th–5th instars in comparison with H_2_O as a control. RNA was extracted from nymphs treated twice with dsRNA-*DcAQP.* Figure 4A shows the presence of the transcript in the non-treated control but not the *DcAQP*-silenced nymphs, indicating the knockdown of gene expression. Furthermore, the effect of *DcAQP* silencing on the nymph mortality (%) of 4th–5th instars of *D. citri* carried out using seven concentrations of dsRNA-*DcAQP* revealed that the nymph mortality (%) increased after the application of dsRNA-*DcAQP* compared to H_2_O and dsRNA-*GFP* (Figure 4B). However, only the 200 ng treatment was statistically different than the controls, and nymph mortality did not show a dose-dependent effect.

### 3.5. DcAQP Silencing Decreases the Survival, Down-Regulates DcAQP and Accumulates Uric Acid in Emerged Adult D. citri

**Figure 5:**
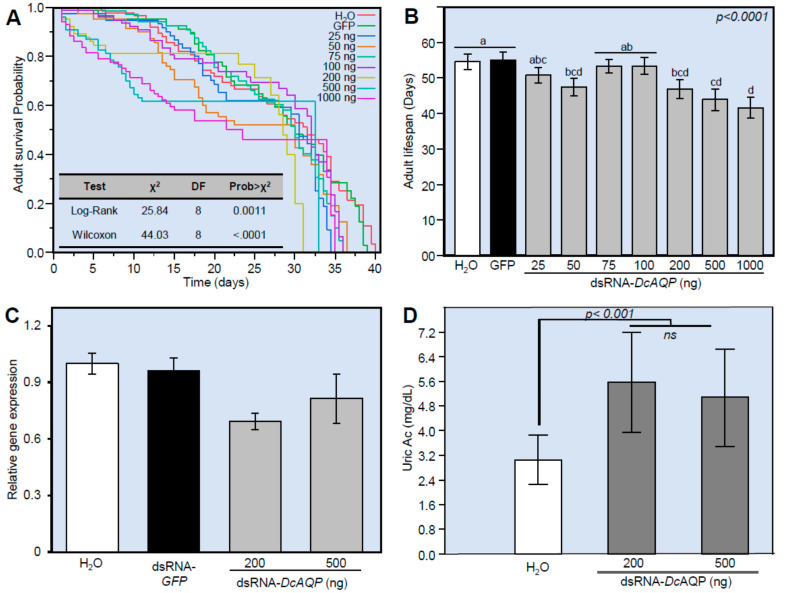
Effect of *DcAQP* silencing on the survival, *DcAQP* expression, and uric acid content of emerged adults of *D. citri.* (**A**) Kaplan–Meier analysis of cumulative survival of *D. citri* adults emerged from treated nymphs after the treatment with different concentrations of dsRNA-*DcAQP* (25, 50, 75, 100, 200, 500 and 1000 ng·µL^−1^) in comparison with RNase-free water and dsRNA-*GFP* as controls. *p* values and χ^2^ of log-rank and Wilcoxon tests (presented in the left corner of the graph) were used for statistical comparisons among the survival curves. (**B**) Lifespans associated with cumulative survival of *D. citri* adults emerged from treated nymphs after the treatment with different concentrations of dsRNA-*DcAQP* (25, 50, 75, 100, 200, 500 and 1000 ng·µL^−1^) in comparison with RNase-free water and dsRNA-*GFP* as controls. (**C**) Relative gene expression of *DcAQP* gene after the treatment with 200 or 500 ng·µL^−1^ dsRNA-*DcAQP* in comparison with RNase-free water and dsRNA-*GFP* as controls. Gene expressions were normalized using actin as a housekeeping gene, and the changes were analyzed with the 2^−ΔΔ*C*^_T_ method. (**D**) Uric acid content of *D. citri* adults emerged from nymphs after the treatment with 200 or 500 ng·µL^−1^ dsRNA-*DcAQP* in comparison with RNase-free water as control. In panels (**B**), (**C**), and (**D**), bars and error bars represent means and SDs, respectively. Different letters indicate statistically significant differences among treatments, while the same letter signifies no significant differences among treatments using Tukey HSD (*p* < 0.05). “ns” signify no significant differences among treatments.

To evaluate the effect of *DcAQP* silencing on the survival of newly emerged adults from dsRNA-*DcAQP*-treated nymphs, we treated insects from the 4th–5th instars with seven concentrations of dsRNA-*DcAQP* (25, 50, 75, 100, 200, 500 and 1000 ng·µL^−1^) and maintained them on citrus to obtain the emerged adults. The Kaplan–Meier analysis of cumulative survival up to 40 days showed that the survival of the emerged adults from dsRNA-*DcAQP*-treated nymphs was significantly lower at all concentrations compared to the controls (H_2_O and dsRNA-*GFP*), and it was not dose-dependent (χ^2^ = 25.84 and 44.03; *p*-value = 0.0011 and <0.0001 for Log-Rank and Wilcoxon tests, respectively) (Figure 5A). Instead, the high concentrations of dsRNA-*DcAQP* (200, 500 and 1000 ng·µL^−1^) but not the low concentrations (25, 75 and 100 ng·µL^−1^) significantly decreased the survival and shortened the lifespan of emerged adults, compared to the mock- and dsRNA-*GFP*-treated controls suggesting a threshold response (Figure 5B).

Furthermore, we determined the transcript level of *DcAQP* approximately 12 days post-treatment (dpt) in the emerged adults from 4th–5th nymph instars treated with the most effective concentrations (200 and 500 ng·µL^−1^). The *DcAQP* gene expression decreased slightly in the emerged adults from nymphs treated with 200 ng·µL^−1^ dsRNA-*DcAQP* (Figure 5C). Moreover, we investigated the effect of *DcAQP* silencing on the uric acid content of the whole body of emerged adults. Although there were no significant differences between the two studied concentrations (200 and 500 ng·µL^−1^ dsRNA-*DcAQP*), both concentrations increased the endogenous levels of uric acid in comparison with the mock control (Figure 5D).

### 3.6. DcAQP Silencing Alters the Body Weight and Length of Adults Emerged from Treated Nymphs

**Figure 6:**
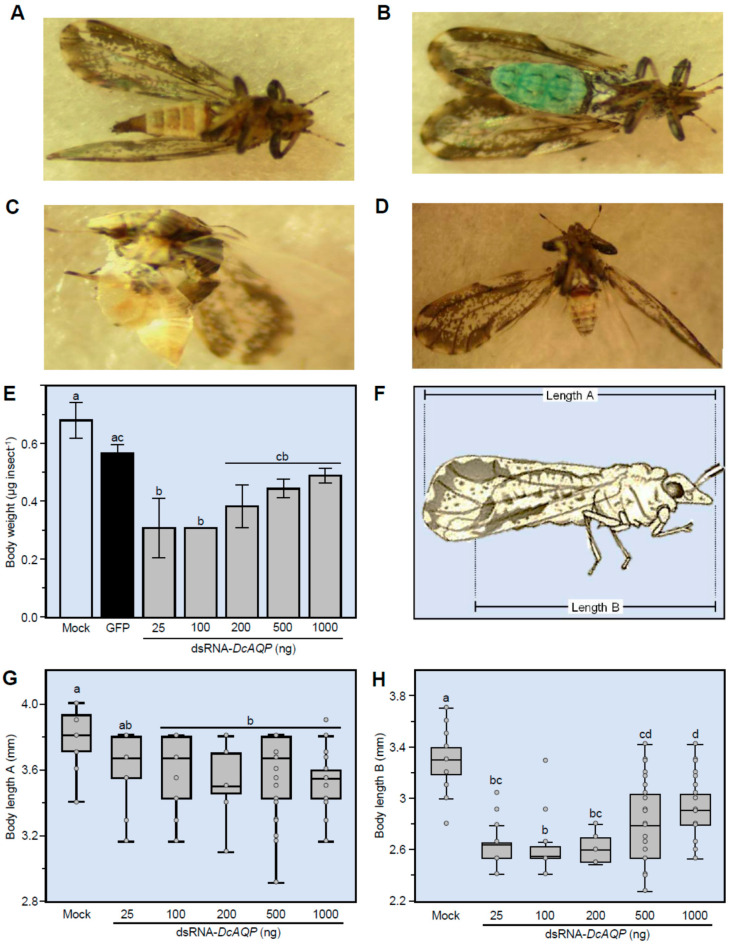
Effect of *DcAQP* silencing on the body weight and length of emerged adults of *D. citri* from treated nymphs. (**A**–**D**) Malformations of emerged adults of *D. citri* from treated nymphs after the treatment with dsRNA-*DcAQP*. (**E**) Body weight of *D. citri* adults emerged from nymphs after the treatment with different concentrations of dsRNA-*DcAQP* (25, 100, 200, 500 and 1000 ng·µL^−1^) in comparison with RNase-free water and dsRNA-*GFP* as controls. Bars and error bars represent means and SDs, respectively. Different letters indicate statistically significant differences among treatments, while the same letter signifies no significant differences among treatments using Tukey–Kramer honestly significant different test (Tukey HSD; *p* < 0.05). (**F**) Graphic illustration represents how the body length was measured. “Length A” covers the distance from the beginning of the head to the end of the wings, whereas “length B” covers the distance from the beginning of the head to the end of the body. (**G**) and (**H**) Body length (“Length A” and “Length B”, respectively) of *D. citri* adults emerged from nymphs after the treatment different concentrations of dsRNA-*DcAQP* (25, 100, 200, 500 and 1000 ng·µL^−1^) in comparison with RNase-free water as control. Horizontal thick lines indicate the medians; black or white dots indicate the means; boxes show the interquartile ranges including 25–75% of the values; whiskers reflect the highest and the lowest value of data. Different letters indicate statistically significant differences among treatments using Tukey HSD (*p* < 0.05).

We observed that *D. citri* treated with the high concentrations of dsRNA-*DcAQP* (more than 200 ng·µL^−1^) had some malformations (reduced body size and incompletely molted) (Figure 6A–D). Therefore, we decided to study the effect of *DcAQP* silencing on body measurements (weight and length). Interestingly, all studied concentrations of dsRNA-*DcAQP* (25, 100, 200, 500 and 1000 ng·µL^−1^) significantly reduced the body fresh weight (μg·insect^−1^) 10 dpt in comparison with both mock- and dsRNA*-GFP*-treated controls (Figure 6E). Furthermore, we measured the body length in two ways: “length A” covers the distance from the beginning of the head to the end of the wings, whereas “length B” covers the distance from the beginning of the head to the end of the body (Figure 6F). Generally, *DcAQP* silencing reduced the body length of emerged adults from treated nymphs (Figure 6G,H). The body length A was significantly decreased 10 dpt with higher concentrations (100, 200, 500 and 1000 ng·µL^−1^ dsRNA-*DcAQP*)*,* but not when nymphs were treated with 25 ng·µL^−1^ (Figure 6G). Likewise, the body length B was significantly decreased at 10 dpt with 25 ng·µL^−1^ dsRNA-*DcAQP* or higher (Figure 6H). These results suggest a body reduction from concentrations as low as 25 ng·µL^−1^ but without affecting the length of the wings.

### 3.7. Application with dsRNA-DcAQP Alters the Metabolite Profile in Adults Emerged from Treated Nymphs

The level of sugars, organic acids, and amino acids were affected in *D. citri* adults treated by dsRNA-*DcAQP*. We detected 41 compounds by gas chromatography-mass spectrometry (GC-MS) through MSTFA derivatization, and 17 compounds through MCF derivatization (Table 2 and Table 3). By MSTFA derivatization, we found significant differences in _L_-alanine, oxalic acid, phosphoric acid, threonine, 2-piperidinecarboxylic acid, β-alanine, xylose, arabinose, putrescine, β-glycerophosphate, citric acid, an unknown sugar (two peaks), glucose (two peaks), *chiro*-inositol, an unknown sugar alcohol 5, glucuronic acid, *myo*-inositol and oleic acid. Interestingly, all compounds that were significantly different increased in the range of 2–3 fold with respect to the control, and none were decreased (except peak 2 of the unknown sugar eluting before glucose). Otherwise, through MCF derivatization, we found that L-phenylalanine and heptadecanoic acid (C17:0) were increased, but _L_-proline, L-glutamic acid, L-histidine and maleic acid were significantly diminished with respect to the control.

## 4. Discussion

Since the earliest identification of the molecular water channel (aquaporin-1 (AQP1)) in red blood cells in the early 1990s, a number of genomic and transcriptomic projects have identified numerous homologous water channel proteins in bacteria, plants, and animals [4,8]. Most biochemical characterizations of aquaporins, however, have been in mammals, and little effort has been made in invertebrates. However, AQP homologues have been found in a wide range of insect genomic databases of which three have been characterized in Hemiptera [10,39]. Nowadays, bioinformatic analysis is a powerful tool to predict protein function by identifying conserved motifs and/or specific domains in protein families [40].

In this work, the results from bioinformatic analysis clearly demonstrated the presence in *DcAQP* sequences of the six transmembrane helices (TM1-6) and five loops (A-E) typical of other aquaporins. Loops B and E are highly symmetrical and contain the canonical Asn-Pro-Ala (NPA) region. Both the N- and C-terminus of the protein are intracellular. These characteristic features comprise the classic ‘hourglass’-shaped model for AQP1 [5,9,12,41]. In fact, these two hallmark regions of *DcAQP* were matched with sequences of *B. tabaci*, *A. pisum*, *M. persicae*, *A. aegypti* and *D. melanogaster* that have been well-characterized as functional water channels. Additionally, the results of the alignment and secondary structure prediction show that the *DcAQP* sequence could belong to class 1 of AQPs subfamily. AQP1 belongs to class 1 and is expressed in the alimentary tracts and Malpighian tubules (insect kidney) in several insects, where it plays an important role for osmoregulation in sap feeders like leafhopper [21], pea aphid [16]; and whitefly [18].

Recently, several roles for AQPs were described in silk moth egg development, including hydration and osmotic swelling of oocytes, and water transport during vitellogenesis [42]. However, in our results, we found that the expression pattern of the aquaporin *DcAQP* transcript in *D. citri* eggs was undetectable in agarose gel after semi-quantitative RT-PCR analysis. This result is possible if the eggs were diapaused or because the AQPs are expressed at different levels in different tissues during insect development, depending on the physiological necessity of the development stage [10,18].

The silencing of target genes through RNAi technique using dsRNA is becoming a powerful tool for insect pest management [30,43,44,45,46]. Effective delivery methods for dsRNA have been developed for different *D. citri* life stages and have allowed the identification of target genes that could be beneficial for controlling this vector. Specifically, these methods include the use of an artificial diet, soaking, and topical feeding [17,31,32,33,47]. In the current study, we tested both soaking and topical feeding for dsRNA delivery to 4th and 5th instar nymphs and found the soaking method to be most efficient for this study. In addition, we saw a significant decrease in *D. citri* fitness only when we applied the dsRNA twice in a 24 h interval through the soaking method. It could be that we obtained these results because successful RNAi depends on several factors including species, the gene targeted, its expression profile, as well as intracellular transport and degradation which is more susceptible when is applied orally or by soaking instead of injection [46,48,49]. For example, cell lines of Lepidoptera were insensitive to dsRNA soaking of targeted housekeeping genes such as *Actin* and coat protein II (Sec23) while cell lines of Coleoptera produced siRNA after soaking in the same dsRNA [49].

It is interesting to note that the mortality measured on day 7 after the last application of dsRNA-*DcAQP* was high and showed a tendency to decrease the survival probability even at low concentrations. However, by the end of the experiment, we considered that there was no impact on the survival parameter. Additionally, the results of the gene expression indicated a total knockdown only 24 h after application of the dsRNA-*DcAQP* in nymphs, and the effect was dramatically reduced on *DcAQP* gene expression when the adults emerged approximately 15 days after treatment. These results indicated that the dsRNA was effective only during the first few days after application. The loss of effect on the *DcAQP* expression could be because the dsRNA is lost or consumed during nymph development, leaving little for suppression in the adults. In our earlier work with *CYP4* genes [50], we also found a transient effect. When dsRNA was applied topically to adult *D. citri*, gene expression was suppressed significantly for 8 dpt [50]. This transient effect was observed in ApAQP1 of pea aphid, where the effect of dsRNA persisted less than a week [16]. It is reasonable to have a transient effect considering that there are several classes of AQPs, and particularly, class 1 has several AQP subfamilies, which could remain functional and could act as a compensatory mechanism.

Homeostasis of water and energy is one of the many critical roles of cellular membranes. Several physiological processes that are dependent on osmotic equilibrium start to fail when the internal steady state is lost [1,11,51]. Therefore, we hypothesize that although the silencing effect on gene expression of *DcAQP* was transient, it was enough to cause a reduction in body size, likely due to dehydration from the effects of high osmotic pressure and the inability to move water through the cell membranes. However, because we did not measure the insect dry weight in our experiments, we cannot confirm dehydration as the cause of reduced body size. We hypothesize that this mass reduction is due to water loss because we silenced AQP gene, which is one of the important mechanisms controlling insect osmotic pressure. The nymphal stage is characterized by voracious feeding behavior [52]. Therefore, silencing *DcAQP* in nymphs disrupts the transport of water, nutrients, metabolites, hormones and body wastes more in nymphs than in adults, which feed less. Furthermore, in the silk moth, *Hyalophora cecropia*, it is essential to keep the osmotic pressure constant in order to allow the diffusion between molting fluid and hemolymph during the molting process [53]. It is possible, then, that the phenotypes found with incomplete molts were due to the effect of silencing *DcAQP*.

Additionally, in newly emerged adults we found an increase of uric acid in comparison with the controls. The accumulation of uric acid in adult *D. citri* emerged from treated nymphs confirms the function of AQPs in *D. citri*. Recent studies revealed that insects store uric acid in the fat body and utilize it in diverse manners such as supplying the nitrogen requirement for egg production in females or as a physiological antioxidant [54,55]. One of the theories proposed for biosynthesis of uric acid is the nucleicolytic pathway, which originates from the nucleic acids derived partly from digested food materials and partly from the processes of tissue maintenance and repair [56]. It is also well known that nucleotides are involved in cellular energy conservation reactions, wherefore their catabolism is tightly regulated [56]. The silencing of *DcAQP* could have provoked poor distribution of oxygen due to osmoregulation disruption and then caused ROS damage in some tissues [57], but this work remains to be investigated. In addition, silencing *DcAQP* may have assisted in the recovery of nucleotides produced by damage tissues and incorporated them into the nucleicolytic pathway with the purpose of storing uric acid for later use. Additionally, in bed bugs, the silencing of *ClAQP1* and *ClGLp1* significantly reduced water and urea excretion after blood feeding [22], so it is possible that the silencing of *DcAQP* in 4th–5th instar nymphs reduced nitrogen excretion resulting in uric acid accumulation

Normal excretion of amino acids in some insects occurs through the Malpighian tubules and through the rectum [56]. We found a significant increase in several amino acids when we measured the metabolites by GC-MS in insects previously treated with dsRNA-*DcAQP*. We observed no effect on sucrose metabolism, indicating that sucrose hydrolase was breaking down the sucrose into glucose and fructose. However, glucose accumulated significantly, which suggests that glucose was not totally integrated into the metabolic pathway of glycolysis and the tricarboxylic acid cycle. The alterations in primary and secondary metabolites in *D. citri* adults emerged from RNAi-treated nymphs demonstrate the impact of silencing of *DcAQP* during the metamorphosis.

## 5. Conclusions

In this first study of silencing aquaporin in *D. citri,* we found significant increases in the nymph mortality, reduction in body fresh weight and length and increases in the uric acid content associated with RNAi-treated *D. citri*. Some of these effects were transient, as was found in our previous work with *CYP4*. The silencing of *DcAQP* could have a stronger, more durable effect in the control of *D. citri* if applied in conjunction with other RNAi gene targets such as *DcSuh* to further enhance disruption of *D. citri* osmoregulation. Future research efforts in this area should include evaluation of ROS damage, measurements of hemolymph osmotic pressure or water content, and *DcAQP* localization to specific tissues to further support the functional roles of aquaporin in *D. citri.* In addition, delivery of *DcAQP* as a plant-incorporated protectant via transgenic citrus or as a field-applied or injected product should be investigated as the current method is limited to laboratory investigations.

## Figures and Tables

**Table 1:** Oligonucleotide primer pairs used in the present study ^a^.

Gene	Primer Name	Primer Orination	Primer SEQUENCE	Amplicon Size	Application
*DcAQP*	*DcAQP*	F:	TTGGTCACGTGAGTGGATGT	306	cDNA fragment amplification
		R:	CTGGCACCGAACCCTTAATA		
	*DcAQP*-T7 ^b^	F:	*TTGGTCACGTGAGTGGATGT	346	dsRNA synthesis
		R:	*CTGGCACCGAACCCTTAATA		
	*DcAQP*SRT-	F:	CCTGCTGTCACCATTGGTCT	234	Semi RT-PCR
		R:	GGCTTCCACCGTTAGAACCA		
	*DcAQP*-RT	F:	CTCGAAGCTGTCACACCAAA	129	q RT-PCR
		R:	GGCTTCCACCGTTAGAACCA		
*gfp*	*gfp*-T7 ^b^	F:	*TTTTCAAGAGTGCCATGCCC	341	dsRNA synthesis
		R:	*TGGTAAAAGGACAGGGCCAT		
	*gfp*-RT	F:	GACGGGACCTACAAGACACG	165	q RT-PCR
		R:	TTGTTTGTCTGCCGTGATGT		
*Actin* ^c^	*Act*-RT	F:	CCCTGGACTTTGAACAGGAA	170	q RT-PCR
		R:	AATCTTGCGGTATCCACGAG		

^a^ Primers were designed by Primer Plus 3 (http://www.primer3plus.com/, accessed on 16 September 2021). ^b^ * T_7_ RNA polymerase promoter fragment: TAATACGACTCACTATAGGG. ^c^ Actin was used as a reference gene for data normalization according to Tiwari, et al. [34].

**Table 2:** Concentrations of different metabolic compounds (ng·insect^−1^) detected in *D. citri* adults after derivatization with MSTFA using gas chromatography-mass spectrometry (GC-MS).

Component	RT ^a^ (min)	Concentration (Means ± std)		*p*-Value ^b^
		Control (*n* = 8*)*	dsRNA-AQP (*n* = 8*)*	
Pyruvic Acid ^d^	4.30	0.732 ± 0.006	0.739 ± 0.007	0.159
L-Alanine ^d^	5.02	0.872 ± 0.320	2.598 ± 0.322	**0.003**
Oxalic acid ^d^	5.13	0.761 ± 0.050	0.916 ± 0.030	**0.002**
Phosphoric acid ^d^	7.13	6.673 ± 3.272	16.214 ± 6.202	**0.028**
L-Isoleucine ^d^	7.43	0.731 ± 0.040	0.722 ± 0.004	0.684
L-Glycine ^d^	7.53	0.838 ± 0.094	0.930 ± 0.217	0.456
L-Threonine ^d^	7.57	0.782 ± 0.063	1.220 ± 0.114	**0.001**
Threose ^d^	10.02	0.728 ± 0.023	0.735 ± 0.025	0.673
2-Piperidinecarboxylic acid ^d^	10.25	3.787 ± 2.064	10.923 ± 1.657	**0.002**
*β*-Alanine ^d^	10.43	0.896 ± 0.154	1.245 ± 0.071	**0.009**
2-Ketoglutaric acid ^d^	10.97	0.801 ± 0.001	0.820 ± 0.018	0.137
Arabinopyranose ^d^	11.63	0.704 ± 0.008	0.698 ± 0.004	0.236
Xylose ^d^	11.99	0.734 ± 0.003	0.814 ± 0.016	**0.001**
Arabinose ^d^	12.09	0.695 ± 0.003	0.715 ± 0.005	**0.001**
Xylose 2 ^d^	12.19	0.692 ± 0.001	0.685 ± 0.001	0.718
Unknown sugar alcohol-1 ^c^	12.25	0.712 ± 0.014	0.713 ± 0.012	0.949
*β*-L-Arabinopyranose ^d^	12.44	0.715 ± 0.015	0.722 ± 0.016	0.551
Arabitol/Xylitol ^e^	12.50	0.714 ± 0.015	0.719 ± 0.015	0.586
Putrescine ^d^	12.64	0.888 ± 0.016	1.123 ± 0.062	**0.002**
Sorbose-1 ^d^	12.91	0.817 ± 0.076	0.804 ± 0.060	0.785
*β*-Glycerophosphate ^d^	12.95	0.785 ± 0.059	1.559 ± 0.081	**0.001**
*α*-D-Mannopyranoside ^d^	13.09	0.708 ± 0.011	0.704 ± 0.007	0.544
Fructofuranose-1 ^d^	13.23	0.713 ± 0.014	0.724 ± 0.019	0.383
Citric acid ^d^	13.53	3.649 ± 0.020	6.388 ± 0.809	**0.002**
Unknown sugar ^c^	14.05	0.902 ± 0.127	1.079 ± 0.047	**0.039**
Unknown sugar ^c^	14.15	0.925 ± 0.021	0.816 ± 0.029	**0.003**
Glucose ^d^	14.37	50.404 ± 1.517	146.530 ± 1.805	**0.001**
*chiro*-Inositol ^d^	14.52	10.980 ± 0.695	30.402 ± 1.805	**0.001**
Galactitol ^d^	14.68	0.725 ± 0.021	0.731 ± 0.066	0.854
Glucose-2 ^d^	14.71	1.542 ± 0.058	2.808 ± 0.269	**0.001**
Mannitol ^d^	14.79	1.245 ± 0.062	1.276 ± 0.073	0.523
*scyllo-*Inositol ^d^	14.86	0.761 ± 0.042	0.793 ± 0.055	0.373
Unknown sugar alcohol-2 ^c^	15.58	0.756 ± 0.037	0.994 ± 0.047	**0.001**
Glucuronic Acid ^d^	15.63	0.721 ± 0.020	1.455 ± 0.108	**0.001**
Palmitoleic Acid ^d^	15.67	0.791 ± 0.061	0.796 ± 0.060	0.914
*myo*-Inositol ^d^	16.24	15.543 ± 0.953	27.154 ± 1.288	**0.001**
Oleic Acid ^d^	17.69	1.796 ± 0.118	6.300 ± 0.647	**0.001**
Stearic Acid ^d^	17.99	0.787 ± 0.095	0.828 ± 0.080	0.535
Unknown sugar alcohol-3 ^c^	20.83	1.022 ± 0.188	0.993 ± 0.145	0.802
Sucrose ^d^	21.58	1.715 ± 0.162	1.881 ± 0.259	0.305
Trehalose ^d^	22.54	109.770 ± 5.943	103.000 ± 3.623	0.167

^a^ Retention times. ^b^ *p*-value < 0.05 (highlighted in bold) indicates statistically significant differences between studied treatments using two-tail *t*-test (*p <* 0.05). ^c^ Detected compounds were tentatively classified into organic groups based on the presence of ion fragments typical of those groups. ^d^ Detected compounds were identified using authentic standards.

**Table 3:** Concentrations of different metabolic compounds (ng·insect^−1^) detected in *D. citri* adults after derivatization with MCF using gas chromatography-mass spectrometry (GC-MS).

Component ^a^	RT ^b^ (min)	Concentration (Means ± std)		*p*-Value ^c^
		Control (*n*= 5)	dsRNA-AQP (*n*= 12)	
L-Alanine	8.96	4.195 ± 0.939	3.871 ± 0.376	0.312
*γ*-Aminobutyric acid	9.38	6.081 ± 0.972	6.008 ± 0.824	0.877
L-Iso-leucine	12.18	0.616 ± 0.231	0.595 ± 0.179	0.853
L-Threonine	12.36	0.590 ± 0.184	0.630 ± 0.068	0.510
L-Proline	12.84	2.034 ± 0.193	1.599 ± 0.120	**0.001**
L-Glutamic Acid	15.37	23.169 ± 5.622	16.735 ± 3.431	**0.015**
L-Methionine	15.55	0.368 ± 0.110	0.403 ± 0.078	0.492
L-Cysteine	15.61	1.219 ± 0.494	1.809 ± 1.479	0.456
L-Phenylalanine	16.89	0.682 ± 0.250	0.991 ± 0.179	**0.011**
L-Histidine	17.67	5.014 ± 0.931	2.618 ± 1.477	**0.009**
L-Tyrosine	18.96	10.354 ± 1.214	10.694 ± 2.160	0.749
Palmitic acid (C16:0)	19.15	0.135 ± 0.020	0.153 ± 0.025	0.174
Heptadecanoic acid (C17:0)	20.14	0.008 ± 0.002	0.0168 ± 0.006	**0.019**
Linoleic acid (C18:2)	20.83	1.042 ± 0.091	1.035 ± 0.230	0.952
Oleic Acid (C18:1)	20.91	1.789 ± 0.164	1.789 ± 0.164	0.676
Stearic acid (C18:0)	21.11	0.148 ± 0.013	0.138 ± 0.015	0.203
Maleic acid	23.45	245.810 ± 66.875	190.551 ± 11.715	**0.020**

^a^ All detected compounds were identified using authentic standards. ^b^ Retention times. ^c^ *p*-value < 0.05 (highlighted in bold) indicates statistically significant differences between studied treatments using two-tail *t*-test (*p* < 0.05).

## Data Availability

Data will be shared upon request to the corresponding authors.

## Supplementary Materials

The following supplementary materials are available online at https://www.mdpi.com/article/10.3390/insects12100931/s1. Table S1: Candidate aquaporin homologs of *D. citri* producing significant alignments with the aquaporin from human, *Homo sapiens* **(***HsAQP1*; accession no. P29972.3). Table S2: Aquaporin gene sequences used in the phylogenetic tree.
