# Supplementary material for: Silencing of Aquaporin Homologue Accumulates Uric Acid and Decreases the Lifespan of the Asian Citrus Psyllid, Diaphorina citri (Hemiptera: Liviidae)
Source: Insects. 2021 Oct 13;12(10):931. doi: 10.3390/insects12100931 (PMC8539622; doi:10.3390/insects12100931)
Supplement: Supplementary file 1 [file insects-12-00931-s001.zip › insects-1403017-supplementary.pdf]

Table S1. Candidate Aquaporin homologs of *D. citri* producing significant alignments with the aquaporin from human, *Homo sapiens* (*HsAQP1*; accession no. P29972.3) [a].

NCBI Database								Psyllid Database (*Diaphorina citri* OGS v2.0 Proteins)						
**Description**	**Accession**	**aa**	**Max Score**	**Total Score**	**Query Cover (%)**	**E value**	**Per. Ident (%)**	**Description**	**Description**	**aa**	**Per. Ident (%)**	**Aln**	**E value**	**Score**
Aquaporin AQPAe.a	XP_008484232.1	270	194	194	84	2e-61	43.86	Aquaporin AQPAe.a 2-RE.	DcitrP097770.1.5	249	43.53	101/232	2e-62	196
								Aquaporin AQPAe.a 2-RC.	DcitrP097770.1.3	249	43.53	101/232	2e-62	196
								Aquaporin AQPAe.a 2-RA.	DcitrP097770.1.1	250	43.53	101/232	2e-62	196
								Aquaporin AQPAe.a 2-RD.	DcitrP097770.1.4	256	43.53	101/232	3e-62	196
								Aquaporin AQPAe.a 2-RB.	DcitrP097770.1.2	256	43.53	101/232	3e-62	196
Aquaporin AQPAe.a	XP_026677945.1	269	166	166	83	2e-50	39.56	Aquaporin 4-like protein 2-RA.	DcitrP039775.1.1	260	39.56	89/225	1e-50	166
								Aquaporin 4-like protein 2-RB.	DcitrP039775.1.2	269	39.56	89/225	2e-50	166
								Aquaporin 4-like protein 2-RA.	DcitrP097795.1.1	269	39.82	88/221	3e-50	166
Aquaporin-4-like	XP_008487274.2	237	103	103	79	1e-26	27.48	Aquaporin AQPAe.a 2-RA.	DcitrP039960.1.1	250	30.63	68/222	1e-24	98.6
								Aquaporin AQPAe.a 2-RA.	DcitrP039965.1.1	292	30.63	68/222	2e-24	99.0
								Aquaporin AQPAe.a 2-RA.	DcitrP039950.1.1	292	30.63	68/222	2e-24	99.0
Aquaporin	AIU99816.1	102	88.2	88.2	33	2e-22	47.25	Aquaporin	DcitrP039815.1.2	129	46.73	50/107	1e-27	103
								Aquaporin	DcitrP039815.1.1	129	46.73	50/107	1e-27	103
Aquaporin AQPAe.a-like	XP_026680357.1	248	76.6	76.6	79	1e-16	29.30	Aquaporin AQPAe.a 2-RA.	DcitrP039960.1.1	250	30.63	68/222	1e-24	98.6
								Aquaporin AQPAe.a 2-RA.	DcitrP039965.1.1	292	30.63	68/222	2e-24	99.0
								Aquaporin AQPAe.a 2-RA.	DcitrP039950.1.1	292	30.63	68/222	2e-24	99.0
Aquaporin-4-like	XP_017303216.2	167	73.2	73.2	71	5e-16	29.29	Aquaporin 4-like protein 2-RA.	DcitrP090745.1.1	242	30.14	63/209	3e-22	92.0
Aquaporin-4-like	XP_008479681.1	178	72.8	72.8	61	7e-16	24.14	-	-	-	-	-	-	-
Neurogenic protein big brain	XP_026681128.1	875	72.0	142	79	2e-14	29.33	Neurogenic protein big brain	DcitrP005045.1.1	119	39.09	43/110	2e-13	65.1
Aquaporin TIP1-2-like	XP_026684712.1	112	53.1	53.1	23	3e-09	41.27	-	-	-	-	-	-	-
Aquaporin-1-like	XP_026686140.1	205	54.3	54.3	30	5e-09	37.04	-	-	-	-	-	-	-
Aquaporin-2-like	XP_008487273.1	184	52.8	52.8	43	2e-08	27.97	-	-	-	-	-	-	-
Intraflagellar transport protein 81 homolog	XP_026682461.1	527	46.2	46.2	15	8e-06	44.19	-	-	-	-	-	-	-

[a] Provided genes have been matched using the protein-protein BLAST (BLASTP 2.8.0+) (Altschul et al., 1997, 2005), based on recent available data in GenBank, National Center for Biotechnology Information website (NCBI, https://www.ncbi.nlm.nih.gov/protein/1-9-2021) and the official gene set v2.0 (OGS-v2.0 proteins) for *D. citri* on citrus greening solutions website (https://citrusgreening.org/organism/Diaphorina_citri/genome/1-9-2021).

Table S2. Aquaporin gene sequences used in the phylogenetic tree

Organism	Accession number and Database
D. citri	XP_008484232.1 NCBI ; A0A1S3DLD8_DIACI UniProt
B. tabaci	B5L019_BEMTA UniProt
M. persicae	A0A0H3XRM3_MYZP UniProt
A. pysum	B8RCD1_ACYPI UniProt
C. quinquefasciatus	B0WQA3_CULQU UniProt
B. tabaciB	A0A1I9WA80_BEMT UniProt
A. mellifera	A0A088A4U5_APIM UniProt
D. busckii	A0A0M4EHN7 UniProt
D. ananassae	B3MDR4 UniProt
C. tarsalis	A0A1Q3FJU2_CULTA UniProt
A. albopictus	A0A182H3E9_AEDAL UniProt
F. bacteriumB	A0A2E7MG94_9FLA UniProt
E. coliB	A0A1E5WVX1_ECOL UniProt
C. trifoliata(2)	A0A060BRL1_9ROS UniProt
C. trifoliata	A0A060BKS6_9ROS UniProt
F. bacterium	A0A2E5ZSN1_9FLA UniProt
E. coli	A0A2B7LP91_ECOL UniProt
D. melanogaster	H6V591_DROME UniProt
A. aegypti	Q9NHW7.2 NCBI
